# Use of Botulinum Toxin as a Treatment of Hemiplegic Shoulder Pain Syndrome: A Randomized Trial

**DOI:** 10.3390/toxins15050327

**Published:** 2023-05-11

**Authors:** Eduardo de Melo Carvalho Rocha, Marcelo Riberto, Rodrigo da Ponte Barbosa, Renan Miguel Porcini Geronimo, Mauricio Menezes-Junior

**Affiliations:** 1 Rehabilitation Service, Faculdade de Ciências Médicas da Santa Casa de São Paulo, São Paulo 01221-021, Brazil; 2 Programa de Pós graduação em Ciências da Saúde Aplicadas ao Aparelho Locomotor, Universidade de São Paulo, Ribeirão Preto 14049-900, Brazil; marceloriberto@gmail.com (M.R.);

**Keywords:** painful shoulder, hemiplegia, spasticity, treatment, botulinum toxin type A, pain, functionality

## Abstract

Objective: The primary objective of this paper is to assess whether the use of 200 units of abobotulinum in the pectoralis major and subscapularis muscles modifies the pain complaint assessed using the visual analog scale in subjects with shoulder pain after the onset of spastic hemiplegia due to cerebrovascular disease when compared to the application of a placebo to the same muscles. Design: A prospective, double-blind, randomized, and placebo-controlled clinical trial study in two different rehabilitation centers. Setting: Two distinct outpatient neurological rehabilitation services. Participants: Patients older than 18 years who were included presented upper limb spasticity resulting from ischemic or hemorrhagic stroke and a diagnosis of Painful Hemiplegic Shoulder Syndrome (PHSS) that was independent of motor dominance. Interventions: Patients were divided into two groups, one of them underwent the application of botulinum toxin (TXB-A) in the pectoralis major and subscapularis muscles, at a total dose of 400 U. Main Outcome Measure: Patients were assessed for a change in pain using the Visual Analog Scale (VAS) for at least 13 mm. Results: An improvement in pain and spasticity levels in both groups, more intense in the toxin group, but without statistical significance. The comparison between the groups showed a reduction in pain by VAS (*p* = 0.52). Conclusions: The use of botulinum toxin in the subscapularis and pectoralis major muscles resulted in a reduction in shoulder pain in spastic hemiplegic patients without statistical significance.

## 1. Introduction

Stroke is a disease with a great impact on the global population, not only because of the immediate consequences of the event and risk of death, but also due to the sequelae originating in the survivors, which influences the quality of life after the disease. Stroke is currently the leading cause of disability in developed countries [1,2,3].

Painful hemiplegic shoulder syndrome (PHSS) is shown in this context as a condition of great relevance due to its high prevalence and impact on quality of life. Thus, PHSS interferes with the function of the upper extremity, impairing daily life activities and appropriate participation in rehabilitation programs [1,4,5,6,7]. Its prevalence varies in the literature, between 16% and 84% [6,7]. Its etiology is still controversial, but several factors have been described as causes such as: rotator cuff injuries, glenohumeral dislocation, impingement syndrome, biceps tendinitis, myofascial pain syndrome, the presence of spasticity and contractures, adhesive capsulitis, nervous compression, and others [8]. In the literature, there are few studies about its risk factors and one may consider the loss of motor control, scapular dyskinesia, glenohumeral subluxation, and spasticity intensity among them [9,10]. The muscles applied with botulinum toxin in PHSS are still a matter of debate, with no consensus on which muscles should be treated. The most frequent pattern of spasticity in PHSS patients is shoulder adduction and internal rotation and the most commonly treated muscles are the pectoralis, subscapularis, teres, and trapezius, among others [11,12,13,14].

## 2. Background

The literature contains few studies to assist therapeutic decision-making by physicians and other professionals involved in rehabilitation programs to justify the use of BTxA as a treatment for PHSS. There is no established guideline on this issue. There are few placebo-controlled studies, none of them using the subscapularis and pectoralis muscles [12,15]. In a recent meta-analysis that was published, only nine studies using botulinum toxin for the treatment of PHSS were included. One of them compared the use of botulinum toxin to an intramuscular placebo using the pectoralis major and the bicep muscles [16], one using only the subscapularis muscle [17], and one using the pectoralis major and teres major [18]. No study has compared the efficacy of the toxin when used on the muscles most often assigned to internal rotation and shoulder adduction (subscapularis and pectoralis major), which justifies this study.

## 3. Aims

To assess whether the use of 200 units of abobotulinum in the pectoralis major and subscapularis muscles modifies the pain complaint, evaluated by the visual analog scale, in subjects with shoulder pain after the onset of spastic hemiplegia due to cerebrovascular disease when compared to the application of a placebo to the same muscles, after 4 months. 

## 4. Specific Objectives

To compare the effect of treating PHSS with BTxA with current standard treatment regarding pain, as assessed using the visual analog scale and McGill pain scale, regarding the articulation range of motion, as assessed using manual goniometry and, regarding functionality, assessed using the Fugl-Meyer upper limb domain test, after 4 months.

## 5. Results

Thirty-five eligible patients were included, and eleven patients were excluded for not achieving the inclusion criteria, not needing to receive the toxin at other points, pain improvement before randomization, and previous shoulder surgeries. Twenty-four patients were randomized, twelve in the therapy group and twelve in the placebo group. After the beginning of the study, two patients were lost in each group. In the therapy group, one patient abandoned the study due to the difficulties imposed by the pandemic but came to the last follow-up and another patient lost contact after the second evaluation. In the placebo group, one was lost due to the impossibility of returning after the first evaluation, and one patient suffered a new stroke and died 2 months after the intervention (placebo).

**Figure 1:**
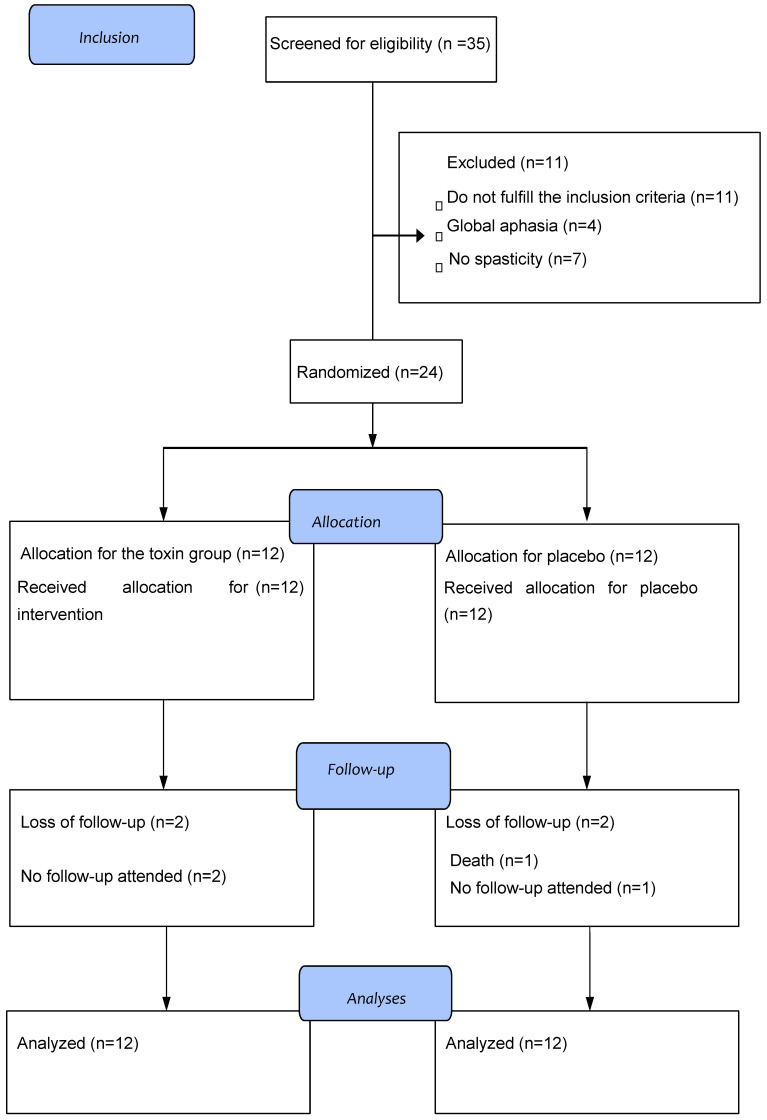
Flow of randomization and patient care.

The randomization and follow-up of the patients are shown in Figure 1.

The distribution of biodemographic description: age, gender, type of stroke, time of stroke, dominance, and affected side is shown in Table 1.

The results described in Table 2, Table 3 and Table 4 demonstrate the pain evaluations between the groups, where there was no significant difference. Table 2 shows the VAS pain scale and its relation to shoulder mobility at the end of the study, which demonstrates any significant changes.

**Figure 2:**
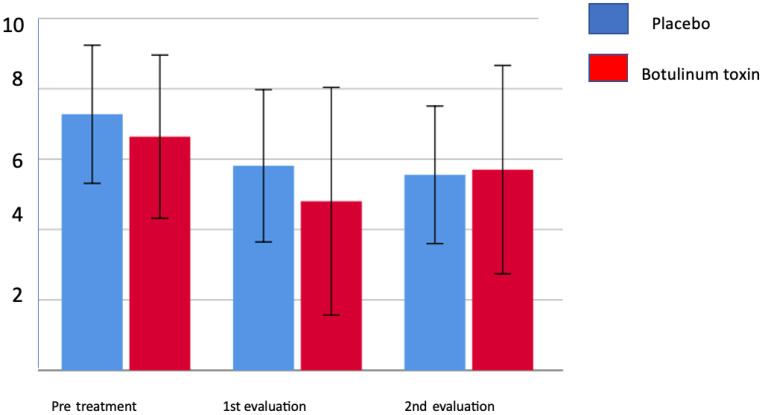
Pain scale (VAS in mm) worse in the last 7 days/treatment group compared to placebo group.

**Figure 3:**
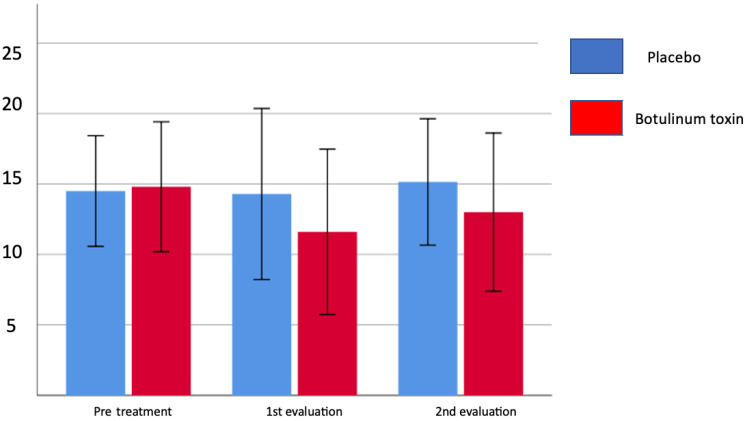
Number of different pain descriptors in the McGill scale/treatment group compared to placebo group.

**Figure 4:**
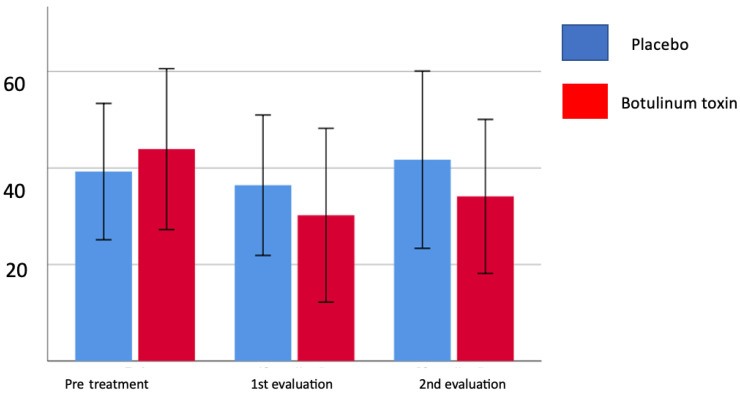
Pain total score by McGill pain scale/treatment group compared to placebo group.

Table 3 shows that the pain index at rest and during active movement before the application was relatively low, and the improvement response happens mainly during the first month, decreasing until the fourth month, but with a reduction in pain levels. Table 4 demonstrates that in the first month, the group treated with botulinum toxin showed a significant reduction in the number of individuals with severe and moderate pain, demonstrating that there may be a benefit from the treatment. It can also be observed that at the end of one month of treatment, both groups showed a reduction in pain, but at the end of four months, the mean value went up again (Figure 2). When the pain is assessed using McGill’s descriptive scale, there was no difference between the groups regarding the total score and the number of descriptors after 4 months, but there was a significant reduction in the number of descriptors after the first month (*p* = 0.012) in the group treated with botulinum toxin. Figure 3 and Figure 4 show the evolution in the McGill scale between the total score and the number of descriptors during treatment shows a more intense reduction in the toxin-treated group, especially in the first month of therapy.

Dividing the groups by the highest pain intensity, we divided patients with mild, moderate, or severe pain in the groups in Table 4.

Figure 2 shows the most intense pain levels reported in the last week before each assessment by the VAS (mm) in both groups. It is observed that at the end of 1 month of treatment, both groups presented pain reduction, but at the end of 4 months, the average value went up again.

Figure 3 and Figure 4 show the evolution in the McGill scale between the total score and number of descriptors during treatment and present a more intense reduction in the group treated with toxin, especially in the first month of therapy. It is important to emphasize that in the group that applied the toxin, the reduction in descriptors in the first month occurred in 50% of patients, mainly in the sensory and affective groups, while in the placebo group, 16.7% had a reduction in the number of descriptors only in the sensory group. A more intense reduction in the toxin-treated group, especially in the first month of therapy, is shown by the evolution in the McGill scale between the total score and the number of descriptors during treatment.

The results described in Table 5 show the variations in spasticity of the shoulder adductor and internal rotator muscles between the groups using the Ashworth scale, where there was also no significant difference, but a more important reduction was observed at month one in the two patterns studied.

The results described in Table 6 show the variations in the range of shoulder movement for abduction and external rotation, active and passive. There was also no significant difference between the groups, but there was a greater gain in the first month in the passive group, the one that received botulinum toxin.

In Table 7, the evolution of the patients functionally evaluated using the Fugl-Meyer scale can be observed, where a greater increase in functionality is noted, especially in the first evaluation in the group that applied the botulinum toxin, with important weight of the sub-item pain and range of motion in this improvement.

## 6. Discussion

The results showed that there was a decrease in the means of pain reported by the patients for abduction and external rotation of the shoulder at rest, during maximum passive and active movement with the use of botulinum toxin, but with no statistically significant difference when compared to the group that received placebo. These observations do not confirm the primary hypothesis of this study, which aimed to demonstrate pain relief as being superior to the placebo and lasting for at least 4 months. This result differs from most studies that showed pain relief gain and shoulder range of motion increase that was superior to the placebo [12,15,18,19].

The mean pain scores at rest were, as expected, the lowest due to the absence of local movement. Active movement despite increasing pain perception was not as intense as during maximum passive movement, which is expected to mobilize more structures and painful areas. Thus, it was observed that, especially in the first evaluation, the pain means in passive movement obtained more expressive reductions (Table 2). The reason we did not find a significant reduction in pain between the groups can be justified by the fact that even the patients in the placebo group received botulinum toxin in other muscle groups, in the distal upper limb, that could improve function and decrease pain. Only two studies showed a greater effect in the placebo group [13,16] and this also differs from this article, which did not show statistical improvement in the toxin group, especially during the first month of follow-up. The use of botulinum toxin for the treatment of other spastic patterns may help to improve pain as a whole [20,21,22]. Another point to be considered is the application of toxin or saline solution in muscles can have an analgesic effect when treating local myofascial pain syndromes, which are also implicated in the genesis of PHSS, associated with the fact that we localize the points through electrostimulation that can by itself decrease local pain [4,23,24]. The results of this study (Table 4) show that there was a reduction in pain severity in more patients in the group applied with the toxin for at least four months, which may indicate that the toxin helps lead to pain improvement for longer periods [12,21].

Table 3 shows that the pain index at rest and during active movement before the application was relatively low, and the improvement response happens mainly during the first month, decreasing until the fourth month, but with a reduction in pain levels. In Table 6, it can be seen that in the first month, the group treated with botulinum toxin showed a significant reduction in the number of individuals with severe and moderate pain, demonstrating that there may be a benefit from the treatment. It can also be observed that, at the end of one month of treatment, both groups showed a reduction in pain, but at the end of four months the mean value went up again (Figure 1). When the pain is assessed using McGill’s descriptive scale, there was no difference between the groups regarding the total score and the number of descriptors after 4 months; however, there was a significant reduction in the number of descriptors after the first month (*p* = 0.012) in the group treated with botulinum toxin. Figure 2 and Figure 3 show the evolution in the McGill scale between the total score and the number of descriptors during treatment, showing a more intense reduction in the toxin-treated group, especially in the first month of therapy. This finding may be related to the fact that the use of botulinum toxin decreases neuropathic pain by local effects on the peripheral nerve and central effects [20,25].

The results presented are in agreement with the response expected by the other methods described in the literature [10,12,26]. We can infer that the application of toxin in other sites away from the shoulder (such as the distal upper limb, and even lower limb muscles) can influence pain mechanisms; in addition, the use of a placebo can contribute to decrease the pain, due to its effect of treating myofascial pain [2].

The choice of subscapularis and pectoralis muscles occurred because both are muscles frequently affected by spasticity after stroke and contribute to internal rotation and adduction of the shoulder, which are factors associated with a higher frequency of painful shoulder syndrome in these patients [10]. In the literature, there are other authors who have used muscles such as the biceps brachial and teres major, among others [13,16,18].

The reason we did not find differences between the spasticity evaluated by the Ashworth scale in the evaluated groups can be explained by the fact that the output spasticity of both groups is low (because they are relatively small muscles) and the improvement can be attributed by the other treatments applied simultaneously (all of our patients were receiving physical therapy and occupational therapy, since before the treatment) [12].

Regarding the range of articular motion of abduction and external rotation of the shoulder, there was also no significant change, as seen in Table 6. This finding coincides with some authors, who show that there are important gains with the use of a placebo also in the range of motion [11,15,27].

As all the patients received toxin application in other areas at the same time in the study, some improvement can also be attributed to functional gain, increased gait, and the therapies performed in association.

We also observed, using the Fugl-Meyer scale, functional gain in the first month of follow-up, but also without significant differences between the groups after four months. This improvement, although partial, shows that the toxin group had a much clearer evolution than among the participants who received the placebo (Figure 4). The previous studies corroborate this finding of a trend towards functional improvement, which demonstrated in their meta-analysis that a large part of the studies that used the Fugl-Meyer had similar responses [12].

The main limiting factors of the study were that its design was to assess pain primarily, and thus the other outcomes studied may not have been strong enough to demonstrate a difference. When assessing the results, we can observe that another factor that can be justified is the large standard deviation of the sample, which might be better controlled in the future by larger samples.

## 7. Conclusions

The use of botulinum toxin in the subscapularis and pectoralis major muscles did not result in a reduction in shoulder pain in spastic hemiplegic patients compared with a placebo solution.

## 8. Method

### 8.1. Study Design

A prospective, with two parallel arms, double-blind, randomized, and placebo-controlled clinical trial study was conducted in two different rehabilitation centers. The study was logged in the Clinical Trials platform under the number NCT04470401. This study did not receive any modification after its registration. This study was funded by the authors.

### 8.2. Subjects

Participants in this study included patients followed up in two different outpatient rehabilitation services, who, agreed to the study procedures and signed the Informed Consent Form (ICF) accepted by these institutions’ research ethics committee. This study followed the recommendations of the Helsinki Declaration and Resolution 196/96 of the National Health Council. The Ethics Committee approved the research in June 2019 under Opinion 3.271.406 Ethic Research Committee (ERC). The patients were recruited from January 2020 to September 2021.

### 8.3. Samples

The sample size calculation was based on the published results [19], where the authors observed a medium difference in pain perception improvement between the BTxA and placebo-treated groups of about 30 mm and a standard deviation of about 20 mm. So, in this study with two parallel arms, 18 participants (9 per group) will be needed, considering the significance level of 5% and the statistical power of 80%, for a minimally significant difference of 13 mm reduction on the Visual Analog Scale. Regarding the possibility of losses in the order of 20%, the total final sample size was 24 subjects, i.e., 12 in each group.

### 8.4. Inclusion Criteria

In this study, the patients who were included presented:

- Spastic motor sequelae in the upper limb resulting from ischemic or hemorrhagic stroke;
- Older than 18 years;
- Diagnosis of PHSS independent of motor dominance;
- Willingness and consent of the patient, family members, and/or responsible caregiver to participate in the study. Thus, everyone was informed about the advantages and disadvantages of the treatment, as well as its risks.

### 8.5. Non-Inclusion Criteria

In this study, the patients who were not included presented:

- Painful condition prior to stroke in the shoulder affected by hemiplegia;
- Previous treatment using TXB-A for painful shoulder conditions;
- Pregnant or nursing women;
- Cognitive impairment that impeded assessment and collaboration with treatment;
- Contraindication to the use of botulinum toxin;
- Joint deformity in the shoulder affected.

### 8.6. Exclusion Criteria

Patients who after inclusion were unable to follow the study for reasons not related to health, such as transport limitations and change of address, among others, or development of clinical situations that prevented attendance at follow-up assessments, such as clinical admissions and states of home restriction, were excluded.

## 9. Assessment Tools

The evaluation consisted of obtaining the participants’ biodemographic and clinical information, followed by the evaluation of the outcome variables immediately before the BTxA injection, as well as after one and four months.

The following assessment tools will be used for this study:

- Demographic characterization by age, gender, origin, time of stroke, use of medications, and previous therapies.
- Visual Analog Scale (VAS): Pain is classified as no pain (0), mild (1–3.9), moderate (4–7.9), and severe (8–10). Pain was assessed with the limb at rest and during active and passive mobilization [28].
- Manual goniometry—consists of evaluating the range of motion of the shoulder in the frontal, vertical, horizontal, external, and internal rotation planes of patients in the sitting position. For the study, the ranges of external rotation and shoulder abduction, both active and passive, were considered [29].
- Modified Asworth Scale (MAS): MAS was applied to the same movements evaluated in the goniometry, in this study in the shoulder internal rotator group and shoulder adductors. Patients scored as 1+ were considered as 1 for calculation and statistical purposes.
- Characterization of pain: For this item, the McGill questionnaire will be used. In each subgroup, the first descriptor scores less than the last, and the descriptor of each subgroup is considered the most intense [30].
- Fugl-Meyer Test (FM): Motor assessment includes measurement of movement, coordination and reflex activity of the shoulder, elbow, wrist, hand, hip, knee, and ankle. This scale has a total of 100 points for normal motor function, where the maximum score for upper extremity is 66 [31].

### 9.1. Procedures

#### 9.1.1. Randomization

The subjects were randomized through a computer program-generated list (www.randomization.com) into 2 groups. The first to undergo BTxA treatment and the second to receive the placebo. The randomization list was drawn up in 6 blocks of 4 subjects to ensure balance of the groups. After, the groups were named A or B by an assistant nurse. 

#### 9.1.2. Blinding

Randomization list of subjects was held by the nursing professional, who was the same person responsible for preparing the medication or placebo administered to the participants at both centers. Placebo used was saline solution (2 mL/200U), with the same coloring and appearance as BTxA. All patients, study evaluators, and physicians who applied the botulinum toxin were totally blind.

### 9.2. Muscle Infiltrations

The muscles selected for this treatment were the pectoralis major and subscapularis muscles, which were each treated with 200 U of abobotulinum at two different points per muscle with the same dosage (100U per point, diluted in 1 mL). The application was guided by electrostimulation.

### 9.3. Statistical

Data were stored in an Excel^®^ for Mac version 16.72 spreadsheet and exported to the statistical analysis software IBM SPSS Statistics^®^ version 23.0 for MAC. Comparison between groups of categorical data was performed using Pearson’s chi-square test. Continuous data were tested for their distribution using the Shapiro–Wilk test. When the distribution was normal, Student t-test was performed, when it was different from this assumption, the Mann–Whitney test was performed. Once most data sets did not have a normal distribution, the intra-group comparison was performed using the Friedman test. When a difference was observed between the assessments in the time follow-up, the repeated measures test, Wilcoxon test, was performed and the Bonferroni correction was applied. A value of *p* ≤ 0.05 was accepted as a statistically significant difference.

In the absence of patient follow-up, the value of the last observation was assigned and loaded into the missing analysis.

## Figures and Tables

**Table 1:** Distribution of biodemographic description: age, gender, type of stroke, time of stroke, dominance, and affected side.

Groups	Therapy	Placebo
*n*	12	12
Age	56.5 (±10.2)	61 (±12.1)
Gender	8 (M) (66.7%)	8 (M) (66.7%)
Stroke	11 ischemic strokes (91.7%)	11 ischemic strokes (91.7%)
Stroke time	26.1 (±36.8)	19.4 (±22.9)
Dominance	10 right-handed/(83.3%)	10 right-handed/(83.3%)
Affected side	7 right (58.3%)	7 right (58.3%)

**Table 2:** Results of VAS pain scales (in mm) and shoulder mobility in abduction and external rotation of the shoulder.

		Toxin Group, *n* = 12		Placebo Group, *n* = 12		
		Mean	SD	Mean	SD	*p* Value (Comparison between Groups)
**Pain active external rotation**						
	Pretreatment	5.73	3.98	3.08	3.58	0.092
	After 1 month	5.59	3.41	3.09	3.48	0.074
	After 4 months	3.83	3.07	3.25	3.77	0.655
**Pain active abduction**						
	Pretreatment	5.64	3.78	3.25	3.77	0.104
	After 1 month	5.32	3.32	3.18	3.63	0.091
	After 4 months	5.92	2.87	3.75	3.89	0.251
**Pain maximum passive external rotation**						
	Pretreatment	7.55	2.88	7.75	1.66	0.495
	After 1 month	6.55	2.84	5.95	3.13	0.642
	After 4 months	7.11	2.65	5.58	3.15	0.169
**Pain maximum passive abduction**						
	Pretreatment	7.55	2.81	8.00	1.35	0.811
	After 1 month	6.45	2.91	5.86	2.92	0.641
	After 4 months	7.65	2.23	6.75	3.05	0.495
**External rotation pain at rest**						
	Pretreatment	2.45	3.62	2.92	3.65	0.765
	After 1 month	2.36	2.80	2.55	3.98	0.747
	After 4 months	2.27	2.45	3.75	4.20	0.513
**Abduction pain at rest**						
	Pretreatment	2.45	3.62	2.92	3.65	0.765
	After 1 month	2.45	2.88	2.45	3.83	0.719
	After 4 months	2.27	2.45	3.58	4.06	0.513

**Table 3:** Results of the VAS scales (in mm) between the groups.

		Toxin Group, *n* = 12		Placebo Group, *n* = 12		
		Mean	SD	Mean	SD	*p* Value (Comparison between Groups)
**VAS—seven days**						
	pretreatment	6.64	2.32	7.28	1.96	0.468
	after 1 month	4.89	3.02	5.84	2.07	0.375
	after 4 months	5.65	2.77	5.61	1.87	0.960
**VAS—at rest**						
	pretreatment	2.66	3.29	3.75	3.03	0.396
	after 1 month	2.61	2.36	2.98	2.87	0.721
	after 4 months	2.54	3.10	4.00	3.27	0.263
**VAS active**						
	pretreatment	6.52	3.23	5.26	3.85	0.384
	after 1 month	4.72	3.24	3.21	3.34	0.262
	after 4 months	4.02	3.13	4.93	3.10	0.477
**VAS maximum passive**						
	pretreatment	7.61	2.10	6.74	2.33	0.338
	after 1 month	6.22	2.23	5.88	2.45	0.720
	after 4 months	6.78	2.32	6.47	2.33	0.742
**VAS (mean)**						
	pretreatment	5.35	1.19	6.24	2.33	0.257
	after 1 month	4.79	2.11	4.39	1.84	0.626
	after 4 months	5.31	1.55	4.79	2.23	0.519

**Table 4:** Division by pain intensity among the groups.

	Toxin Group, *n* = 12			Placebo Group, *n* = 12		
Pain Level	Start	1st Assessment	2nd Assessment	Start	1st Assessment	2nd Assessment
Mild (0–<4)	0 (zero)	4 (33.3%)	1 (8.3%)	0 (zero)	2 (16.7%)	1 (8.3%)
Moderate (4–<8)	8 (66.7%)	6 (50%)	9 (75%)	6 (50%)	8 (66.7%)	8 (66.7%)
Severe (8–10)	4 (33.3%)	2 (16.7%)	2 (16.7%)	6 (50%)	2 (16.7%)	3 (25%)

**Table 5:** Results of the spasticity variation using the Ashworth scale between the groups.

		Toxin Group, *n* = 12		Placebo Group, *n* = 12		
		Mean	SD	Mean	SD	*p* Value (Comparison between Groups)
**Ashworth adduction**						
	1	1.92	1.08	1.92	0.79	0.830
	2	1.18	0.60	1.55	0.82	0.234
	3	1.58	1.08	1.50	0.80	0.832
**Ashworth internal rotation**						
	1	1.83	1.19	1.92	0.79	0.739
	2	1.18	0.60	1.64	0.81	0.123
	3	1.50	1.24	1.58	1.08	0.863

**Table 6:** Results of shoulder range of motion variation (ROM) for abduction and external rotation, active and passive, in degrees.

		Toxin Group, *n* = 12		Placebo Group, *n* = 12		
		Mean	SD	Mean	SD	*p* Value (Comparison between Groups)
**ROM active external rotation**						
	1	20.92	25.38	9.17	15.79	0.229
	2	21.00	27.35	7.17	10.31	0.253
	3	20.42	26.33	12.83	18.71	0.593
**ROM passive external rotation**						
	1	34.42	21.92	40.83	26.01	0.136
	2	43.92	18.99	49.67	29.05	0.543
	3	43.00	16.18	48.92	31.72	0.706
**ROM active abduction**						
	1	47.17	32.65	34.83	31.34	0.580
	2	50.50	39.37	38.33	39.04	0.361
	3	46.67	38.81	38.42	35.46	0.677
**ROM passive abduction**						
	1	73.58	18.70	86.25	15.49	0.361
	2	79.83	20.77	74.75	35.34	0.815
	3	79.58	23.11	79.25	29.37	0.977

**Table 7:** Results of the Fugl-Meyer scale variation for upper limbs divided into sub-items between the groups.

		Toxin Group, *n* = 12		Placebo Group, *n* = 12		
		Mean	SD	Mean	SD	*p* Value (Comparison between Groups)
**Fugl-Meyer—Passive motivity and pain**						
	1	25.38	11.54	25.67	10.48	0.950
	2	31.96	7.39	27.37	12.69	0.275
	3	31.58	9.36	26.64	12.35	0.269
**Fugl-Meyer—Sensitivity**						
	1	10.38	2.84	11.25	2.09	0.399
	2	10.88	3.63	12.43	3.09	0.267
	3	9.92	3.18	12.17	3.16	0.090
**Fugl-Meyer—Upper limb motor function**						
	1	16.15	16.35	15.08	12.38	0.856
	2	18.26	17.74	12.84	10.95	0.414
	3	17.77	15.28	16.77	13.46	0.864
**Fugl-Meyer—Coordination/Speed**						
	1	1.31	1.75	2.67	2.15	0.095
	2	3.08	2.59	6.62	8.89	0.249
	3	2.38	1.98	3.50	2.84	0.264
**Fugl-Meyer—Total**						
	1	54.85	28.94	55.33	18.29	0.961
	2	63.81	18.39	48.46	28.09	0.117
	3	60.38	24.17	56.79	21.14	0.697

## Data Availability

All the research data are with the principal author and are protect to maintain participants privacy.

## Abbreviations

PHSS	Painful Hemiplegic Shoulder Syndrome
VAS	Visual Analog Scale
BtxA/TBA	Botulinum toxin/Abobotulinum
ICF	Informed Consent Form
ERC	Ethic Research Committee
MAS	Modified Asworth Scale
FM	Fugl-Meyer Test
ROM	Range of Motion

## References

[1] Murie-Fernández M., Carmona Iragui M., Gnanakumar V., Meyer M., Foley N., Teasell R. (2012). Painful hemiplegic shoulder in stroke patients: Causes and management. Neurologia.

[2] Oliveira e Silva C., Riberto M., Battistella L.R. (2000). Avaliação da dor no ombro em paciente com acidente vascular cerebral. Acta Fisiátrica.

[3] de Oliveira G.M.M., Brant L.C.C., Polanczyk C.A., Biolo A., Nascimento B.R., Malta D.C., de Fatima Marinho de Souza M., Soares G.P., Xavier G.F., Machline-Carrion M.J. (2020). Cardiovascular Statistics—Brazil 2020. Arq. Bras. Cardiol..

[4] Allen Z.A., Shanahan E.M., Crotty M. (2010). Does suprascapular nerve block reduce shoulder pain following stroke: A double-blind randomised controlled trial with masked outcome assessment. BMC Neurol..

[5] Kalichman L., Ratmansky M. (2011). Underlying pathology and associated factors of hemiplegic shoulder pain. Am. J. Phys. Med. Rehabil..

[6] Lee J.A., Park S.W., Hwang P.W., Lim S.M., Kook S., Choi K.I., Kang K.S. (2012). Acupuncture for shoulder pain after stroke: A systematic review. J. Altern. Complement. Med..

[7] Walsh K. (2001). Management of shoulder pain in patients with stroke. Postgrad. Med..

[8] Wilson R.D., Chae J. (2015). Hemiplegic Shoulder Pain. Phys. Med. Rehabil. Clin. N. Am..

[9] Kumar P. (2019). Hemiplegic shoulder pain in people with stroke: Present and the future. Pain Manag..

[10] Anwer S., Alghadir A. (2020). Incidence, Prevalence, and Risk Factors of Hemiplegic Shoulder Pain: A Systematic Review. Int. J. Environ. Res. Public. Health.

[11] Pedreira G., Cardoso E., Melo A. (2008). Botulinum toxin type A for refractory post-stroke shoulder pain. Arq. Neuropsiquiatr..

[12] Xie H.M., Guo T.T., Sun X., Ge H.X., Chen X.D., Zhao K.J., Zhang L.N. (2021). Effectiveness of Botulinum Toxin A in Treatment of Hemiplegic Shoulder Pain: A Systematic Review and Meta-analysis. Arch. Phys. Med. Rehabil..

[13] Kasapoğlu-Aksoy M., Aykurt-Karlıbel İ., Altan L. (2020). Comparison of the efficacy of intramuscular botulinum toxin type-A injection into the pectoralis major and the teres major muscles and suprascapular nerve block for hemiplegic shoulder pain: A prospective, double-blind, randomized, controlled trial. Neurol. Sci..

[14] Gomes A.L.S., de Mello F.F., Neto J.C., Benedeti M.C., Modolo L.F.M., Riberto M. (2019). Can the positions of the spastic upper limb in stroke survivors help muscle choice for botulinum toxin injections?. Arq. Neuro-Psiquiatr..

[15] Singh J.A., Fitzgerald P.M. (2011). Botulinum toxin for shoulder pain: A cochrane systematic review. J. Rheumatol..

[16] Kong K., Neo J., Chua K.S.G. (2007). A randomized controlled study of botulinum toxin A in the treatment of hemiplegic shoulder pain asociated with spasticity. Clin. Rehabil..

[17] Yelnik A.P., Colle F.M., Bonam I.V. (2003). Treatment of pain and limited movement of the shoulder in hemiplegic patients with botulinum toxin a in the subscapular muscle. Eur. Neurol..

[18] Marciniak C.M., Harvey R.L., Gagnon C.M., Duraski S.A., Denby F.A., McCarty S., Bravi L.A., Polo K.M., Fierstein K.M. (2012). Does Botulinum Toxin Type A Decrease Pain and Lessen Disability in Hemiplegic Survivors of Stroke with Shoulder Pain and Spasticity? A Randomized, Double-Blind, Placebo-Controlled Trial. Am. J. Phys. Med. Rehabil..

[19] Marco E., Duarte E., Vila J., Tejero M., Guillen A., Boza R., Escalada F., Espadaler J.M. (2007). Is botulinum toxin type A effective in the treatment of spastic shoulder pain in patients after stroke? A double-blind randomized clinical trial. J. Rehabil. Med..

[20] Ranoux D., Attal N., Morain F., Bouhassira D. (2008). Botulinum toxin type a induces direct analgesic effects in chronic neuropathic pain. Ann. Neurol..

[21] Gracies J.M., Brashear A., Jech R., McAllister P., Banach M., Valkovic P., Walker H., Marciniak C., Deltombe T., Skoromets A. (2015). Safety and efficacy of abobotulinumtoxinA for hemiparesis in adults with upper limb spasticity after stroke or traumatic brain injury: A double-blind randomised controlled trial. Lancet Neurol..

[22] Shaw L., Rodgers H., Price C., van Wijck F., Shackley P., Steen N., Barnes M., Ford G., Graham L., BoTULS investigators (2010). BoTULS: A multicentre randomised controlled trial to evaluate the clinical effectiveness and cost effectiveness of treating upper limb spasticity due to stroke with botulinum toxin type A. Health Technol. Asssess.

[23] Bai Q. (2012). Clinical observation on post-stroke shoulder pain treated with balance acupuncture. Zhongguo Zhen Jiu.

[24] Roosink M., Van Dongen R.T., Buitenweg J.R., Renzenbrink G.J., Geurts A.C., Ijzerman M.J. (2012). Multimodal and widespread somatosensory abnormalities in persistent shoulder pain in the first 6 months after stroke: An exploratory study. Arch. Phys. Med. Rehabil..

[25] Roosink M., Renzenbrink G.J., Geurts A.C., Ijzerman M.J. (2012). Towards a mechanism-based view on post-stroke shoulder pain: Theoretical considerations and clinical implications. Neurorehabilitation.

[26] Dyer S., Mordaunt D.A., Adey-Wakeling Z. (2020). Interventions for Post-Stroke Shoulder Pain: An Overview of Systematic Reviews. Int. J. Gen. Med..

[27] de Boer K.S., Arwert H.J., de Groot J.H., Meskers C.G., Mishre A.D., Arendzen J.H. (2008). Shoulder pain and external rotation in spastic hemiplegia do not improve by injection of botulinum toxin A into the subscapular muscle. J. Neurol. Neurosurg. Psychiatry.

[28] Heller G.Z., Manuguerra M., Chow R. (2016). How to analyze the Visual Analogue Scale: Myths, truths and clinical relevance. Scand. J. Pain..

[29] DeLisa J.A., Gans B.M. (2002). Tratado de Medicina de Reabilitação—Princípios e Prática.

[30] de Mattos Pimenta C.A., Teixeira M.J. (1996). Manoel JacobsenQuestionário de dor McGill: Proposta de adaptação para a língua portuguesa. Revista da Escola de Enfermagem da USP.

[31] Fugl-Meyer A.R., Jääskö L., Leyman I., Olsson S., Steglind S. (1975). The post-stroke hemiplegic patient. 1. a method for evaluation of physical performance. Scand. J. Rehabil. Med..

